# The ATP-Binding Cassette Transporter *ABCB19* Regulates Postembryonic Organ Separation in *Arabidopsis*

**DOI:** 10.1371/journal.pone.0060809

**Published:** 2013-04-01

**Authors:** Hongtao Zhao, Lei Liu, Huixian Mo, Litao Qian, Ying Cao, Sujuan Cui, Xia Li, Ligeng Ma

**Affiliations:** 1 Laboratory of Molecular Cell Biology, Hebei Normal University, Shijiazhuang, Hebei, China; 2 College of Life Sciences, Capital Normal University, Beijing, China; 3 State Key Laboratory of Plant Cell and Chromosome Engineering, Center of Agricultural Resources, Institute of Genetics and Developmental Biology, Chinese Academy of Sciences, Shijiazhuang, Hebei, China; Centrum Wiskunde & Informatica (CWI) & The Netherlands Institute for Systems Biology, The Netherlands

## Abstract

The phytohormone auxin plays a critical role in plant development, including embryogenesis, organogenesis, tropism, apical dominance and in cell growth, division, and expansion. In these processes, the concentration gradient of auxin, which is established by polar auxin transport mediated by PIN-FORMED (PIN) proteins and several ATP-binding cassette/multi-drug resistance/P-glycoprotein (ABCB/MDR/PGP) transporters, is a crucial signal. Here, we characterized the function of ABCB19 in the control of *Arabidopsis* organ boundary development. We identified a new *abcb19* allele, *abcb19-5*, which showed stem-cauline leaf and stem-pedicel fusion defects. By virtue of the DII-VENUS marker, the auxin level was found to be increased at the organ boundary region in the inflorescence apex. The expression of *CUP-SHAPED COTYLEDON2* (*CUC2*) was decreased, while no obvious change in the expression of *CUC3* was observed, in *abcb19*. In addition, the fusion defects were greatly enhanced in *cuc3 abcb19-5*, which was reminiscent of *cuc2 cuc3*. We also found that some other organ boundary genes, such as *LOF1*/*2* were down-regulated in *abcb19*. Together, these results reveal a new aspect of auxin transporter *ABCB19* function, which is largely dependent on the positive regulation of organ boundary genes *CUC2* and *LOFs* at the postembryonic organ boundary.

## Introduction

Throughout the lifespan of most higher plants, new organs are initiated continuously from pluripotent cells in the shoot apical meristem. This essential process is associated with the establishment of boundaries separating the newly formed organs from adjacent tissues [1]. Such boundaries are composed of a specialized group of saddle-shaped cells that are morphologically different from the adjacent cells [2]. The unique shape of these cells is attributed to elongation along the organ boundary, contraction along the axis perpendicular to the boundary, and cell division leading to a new cell wall parallel to the boundary [2]–[4]. These boundaries emerge at the early stage of primordia initiation, and their positions are determined by signals from the central region of the meristem [1], [2], [5]. The boundaries act as a barrier to separate and maintain different cell types [1], and, when localized at the base of leaves, they have the potential to produce axillary meristems, which contribute greatly to the overall architecture of plants [6].

In *Arabidopsis*, a number of genes with a boundary-specific expression pattern have been identified. Among them, *CUP-SHAPED COTYLEDON* (*CUC*) genes are well-known NAC domain-containing transcription factors [7]-[9]. *CUC1*, *CUC2*, and *CUC3* participate redundantly in embryonic meristem formation and cotyledon separation [7], [8]. *CUC2* and *CUC3* play a significant role in the separation of postembryonic organs, including rosette leaves, stems, and pedicels [8]. It has also been reported that two MYB domain-containing transcription factors, *LATERAL ORGAN FUSION1 and LATERAL ORGAN FUSION2* (*LOF1* and *LOF2*), which are specifically expressed at organ boundaries, play critical roles in lateral organ separation [6]. A number of other boundary-specific genes, including *JAGGED LATERAL ORGANS* (*JLO*) [10], *LATERAL SUPPRESSOR* (*LAS*) [11], [12], *BLADE ON PETIOLE* (*BOP*) [13], [14], *REGULATORS OF AXILLARY MERISTEMS* (*RAX*) [15], and *LATERAL ORGAN BOUNDARIES* (*LOB*) family genes [16], [17], have been shown to be involved in embryonic and/or postembryonic boundary specification.

Several lines of evidence show that auxin plays a significant role in organ patterning and boundary establishment by controlling *CUC* gene expression [18]–[21]. Mutations in the putative auxin efflux carrier *PIN1* produce naked inflorescence stems resulting from the ectopic expression of *CUC2* at a ring-like domain characterized by primordia-specific gene expression [18]. *PINOID* (*PID*) and *ENHANCER OF PINOID* (*ENP*) regulate PIN1 localization and function to promote cotyledon initiation bilaterally by preventing *CUC1*, *CUC2*, and *STM* from expanding to the primordia during embryonic development [20], [21]. *MONOPTEROS* (*MP*)/*AUXIN RESPONSE FACTOR5* (*ARF5*), a transcriptional activator of auxin signaling, participates with *PIN1* in cotyledon separation through the partial regulation of *CUC2* [19]. Together, these results suggest a relationship among auxin, auxin transporters, and the regulation of *CUC* expression in the process of organ patterning and boundary establishment [2].

The auxin concentration gradient is a crucial signal during plant development that is established by polar auxin transport [22], [23]. Two protein families, PIN-FORMED (PINs) efflux carriers and ATP-binding cassette/multi-drug resistance/P-glycoprotein (ABCB/MDR/PGP) transporters, are involved in auxin efflux [23]–[26]. PIN encodes a 67-kilodalton protein with similarity to bacterial and eukaryotic carrier proteins [23]. There are eight members of the *PIN* family in the *Arabidopsis* genome [22]. As described above, *pin-formed1* (*pin1*) was first characterized by needle-like inflorescence stems [23]. *pin1* also exhibits defects in vascular patterning, organogenesis, and phyllotaxis [23], [27], [28]. Physiological studies performed to date *in planta* and/or heterologous systems have demonstrated that at least five *PINs* act as a rate-limiting step in cellular auxin efflux. And consistent with their role as auxin polar transporters, some of the PIN proteins display polar localization, especially in embryonic development and organogenesis, although some are distributed without prominent polarity in certain tissues (for a review, see [22]).

In *Arabidopsis*, *ABCB1*/*PGP1* and *ABCB19*/*PGP19*/*MDR1*, like *PINs*, have been shown to be involved in auxin transport in both plant and heterologous systems [24], [25], [29]–[32]. ABCB1/ABCB19, together with the PIN family of proteins, is involved in auxin efflux [33], [34]. ABCB1/ABCB19 and PINs are co-localized in certain tissues, where they interact [33]. These ABCB-PIN protein interactions enhance the efficiency of auxin transport and substrate/inhibitor specificities when co-expressed in a heterologous system [33]. ABCB19 also stabilizes PIN1 in membrane microdomains [35]. ABCB1 and ABCB19 interact with FKBP-like protein TWISTED DWARF 1(TWD1), and their co-expression enhances auxin export in HeLa cells [36], [37]. TWD1 is also necessary for the localization of ABCB19 [38].

ABCB1 and ABCB19 contribute to long-distance basipetal auxin transport in the seedling apex, upper inflorescence stem and root hair cells [24], [25], [29], [32], [39]; moreover, they function in auxin retention in the stele of the root [33]. Mutations in *abcb19* produce several defects, including epinastic cotyledons and first true leaves, curled and wrinkled rosette leaf margins, and slight waviness in the hypocotyl of etiolated seedlings [24]. Lesions in *ABCB1*, the closest relative of *ABCB19*, produce no morphological differences from wild type [24]. However, the *abcb1 abcb19* displays more severe defects than *abcb19* [24]. *ABCB1* and *ABCB19* also participate in photomorphogenesis [32], [40]. Further, *ABCB19* functions in gravitropism and phototropism [29], [41], [42].

Here, we identified a new allele of *abcb19*, named *abcb19-5*, which shows organ fusion defects in addition to the phenotypes already described for *abcb19* [24]. *CUC2* was down-regulated in *abcb19* and *cuc3* greatly enhanced the organ fusion phenotype of *abcb19-5*, reminiscent of the *cuc2 cuc3*. Further more, some other organ boundary genes were also down-regulated in *abcb19*. Our results reveal a new function for the auxin transporter *ABCB19*.

## Results

### *ABCB19* is necessary for organ separation at stem-cauline leaf and -pedicel junctions in *Arabidopsis*

**Figure 1:**
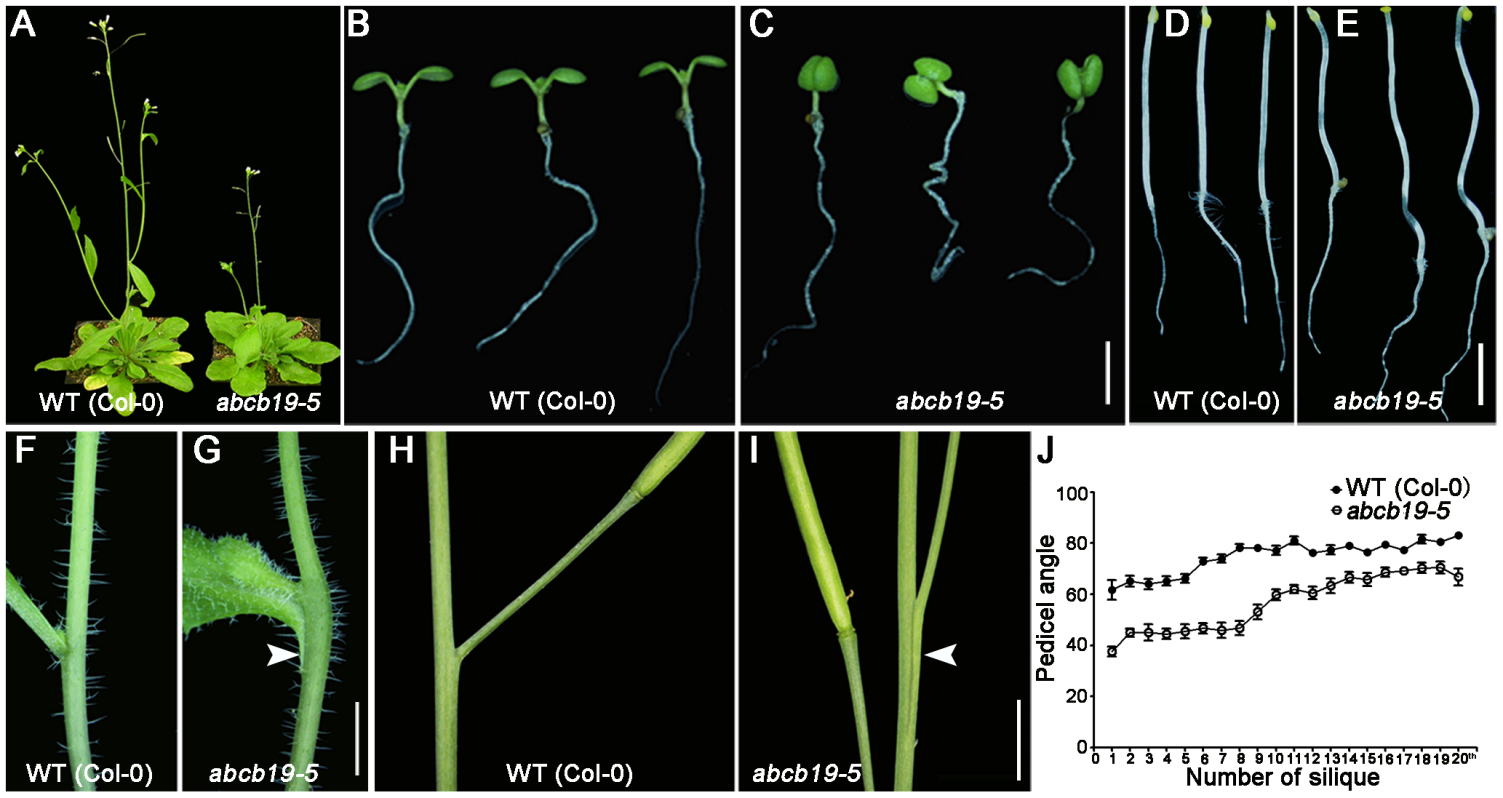
*abcb19-5* exhibits pleiotropic phenotypes. **A:** *abcb19-5* flowers later than wild-type (WT). **B** and **C:** Eight-day-old wild-type and *abcb19-5* plants. *abcb19-5* exhibits epinastic cotyledons and wavy roots. **D** and **E:** Four-day-old seedlings grown in the dark. The hypocotyls of *abcb19-5* are wave-shaped, while those of WT are straight. **F** and **G:** Stem-cauline leaf junctions in WT and *abcb19-5*. *abcb19-5* shows a stem-leaf fusion phenotype. **H** and **I:** Stem-pedicel junctions in WT and *abcb19-5*. The first several pedicels were fused with the main stem in *abcb19-5*. The arrowheads in **G** and **I** indicate the fusion sites. **J** Pedicel angle of the first 20 siliques in WT and *abcb19-5*. For each silique position, 10 samples were photographed and the angles were analyzed using Image J. Scale bar = 2.5 mm in **C** and **E**, and 5 mm in **G** and **I**.

To characterize novel components in flowering time control, we screened a T-DNA insertion mutant library and identified a mutant with a delay in the transition to flowering (Figure 1A). In addition, the mutant exhibited epinastic cotyledons and wavy roots and hypocotyls at the seedling stage (Figure 1B-E). Furthermore, organ fusion defects occurred at both stem-cauline leaf junctions (the abnormal growth of the proximal part of the cauline leaf fused with the stem) (Figure 1F and G) and stem-pedicel junctions (Figure 1H and I). This fusion, which was seen on the primary and secondary branches, was most obvious on rosette branches. The stem-cauline leaf fusions caused bending of the stem (Figure 1G). Stem-pedicel fusions were more obvious for the first several siliques, and, as a result, the angle between the stem and pedicel was significantly reduced for the first eight siliques (Figure 1J).

**Figure 2:**
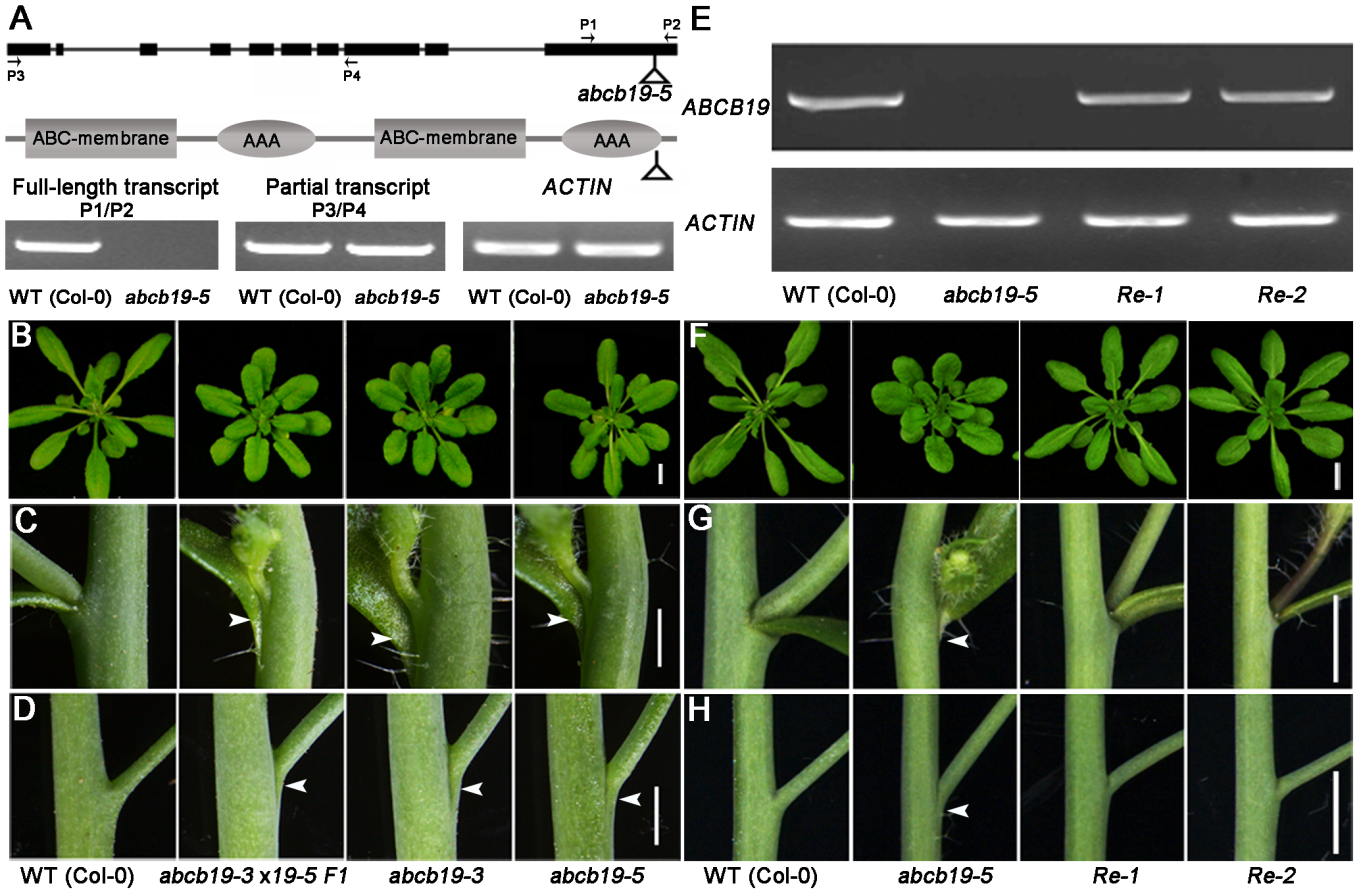
*abcb19-5* is a new T-DNA insertion allele of *ABCB19*. **A:** The upper model shows the gene structure of *ABCB19*. The filled black boxes and lines represent exons and introns, respectively. The lower model shows the protein structure of ABCB19. The protein domain information was analyzed at http://smart.embl-heidelberg.de. The gray boxes and ellipses represent the transmembrane domains and ATP-binding domains in ABCB19, respectively. P1, P2, P3 and P4 are primers used in transcript identification. TAIL-PCR revealed that the T-DNA insertion in *abcb19-5* was in the last exon and the ATP-binding domain in *ABCB19* (shown by triangles). The electrophoretic image shows that *abcb19-5* expressed a partial transcript (P3/P4) rather than the full-length transcript (P1/P2). Rosette leaves (**B**), leaf-stem fusion (**C**), and stem-pedicel fusion defects (**D**) in F1 plants of *abcb19-5*×*abcb19-3*, *abcb19-3* (*mdr1-3*), and *abcb19-5*. **E**-**H** Transgenic complementation of *abcb19-5* by CaMV *35S::ABCB19*. **E** RNA expression level of *abcb19-5* in two transgenic recovered plants (*Re-1* and *-2* are two representative recovered lines). The upper and lower panels represent *ABCB19* and *ACTIN*, respectively. **F** The rosette leaf shape was complemented by *ABCB19*. **G** and **H** Restoration of the stem-cauline leaf and stem-pedicel fusion defects. Arrowheads indicate the fusion sites. Scale = 10 mm in **B** and **F**, and 2 mm in **C**, **D**, **G**, and **H**.

TAIL-PCR was performed to obtain the flanking sequence of the T-DNA left border. Sequencing of the PCR products showed that the adjacent gene was the auxin transporter *ABCB19/PGP19/MDR1*, and that the insertion was in the last exon (Figure 2A). No full-length transcript was detected for this new allele, which was named *abcb19-5*; however, a partial transcript was present (Figure 2A).

To confirm that the mutation of *abcb19* was responsible for these developmental defects, the T-DNA insertion allele *abcb19-3* (*mdr1-3*) was obtained [24]. We found that *abcb19-3* behaved very similar to *abcb19-5*; *abcb19-3* showed the same fusion phenotype (Figure 2B, C, and D) in addition to the other phenotypes described above (data not shown). F1 plants produced by crossing *abcb19-5* with *abcb19-3* also behaved like both of the parents (Figure 2B, C, and D). Moreover, these defects, including epinastic cotyledons, rosette leaf shape, and stem-cauline leaf and -pedicel fusion defects, were successfully rescued by the transformation of *ABCB19* into *abcb19-5* (Figure 2E-H). These results demonstrate that *abcb19-5* is a new allele of *abcb19*. Given that there is still no study about *ABCB19* in organ separation, we focused on the organ fusion phenotype of the mutant.

### Auxin distribution is altered by *ABCB19* mutation

It was reported that *ABCB19* is required for the basipetal auxin transport out of the shoot apex of seedling and inflorescence [24], and that loss of *ABCB19* function increased auxin retention in the apical tissues of seedling by quantification of endogenous IAA levels and radiotracer studies [42]. Due to the organ separation defects of *abcb19*, we are curious about the endogenous auxin level at the organ boundary region in *abcb19*. However, as a result of the auxin distribution gradient, with levels being highest in the primordia and lowest in the organ boundaries [1], it is difficult to analyze the alteration of auxin levels at the site of organ fusion using the auxin-responsive marker *DR5::GUS/GFP*. Fortunately, the DII-VENUS (termed domain II fusion with fast maturating variant of YFP, VENUS) marker is more sensitive than *DR5::GUS/GFP*, images of which are like a photographic negative of auxin levels [43]–[45]. And there are strong signals at the organ boundary at the inflorescence apical region [43].

**Figure 3:**
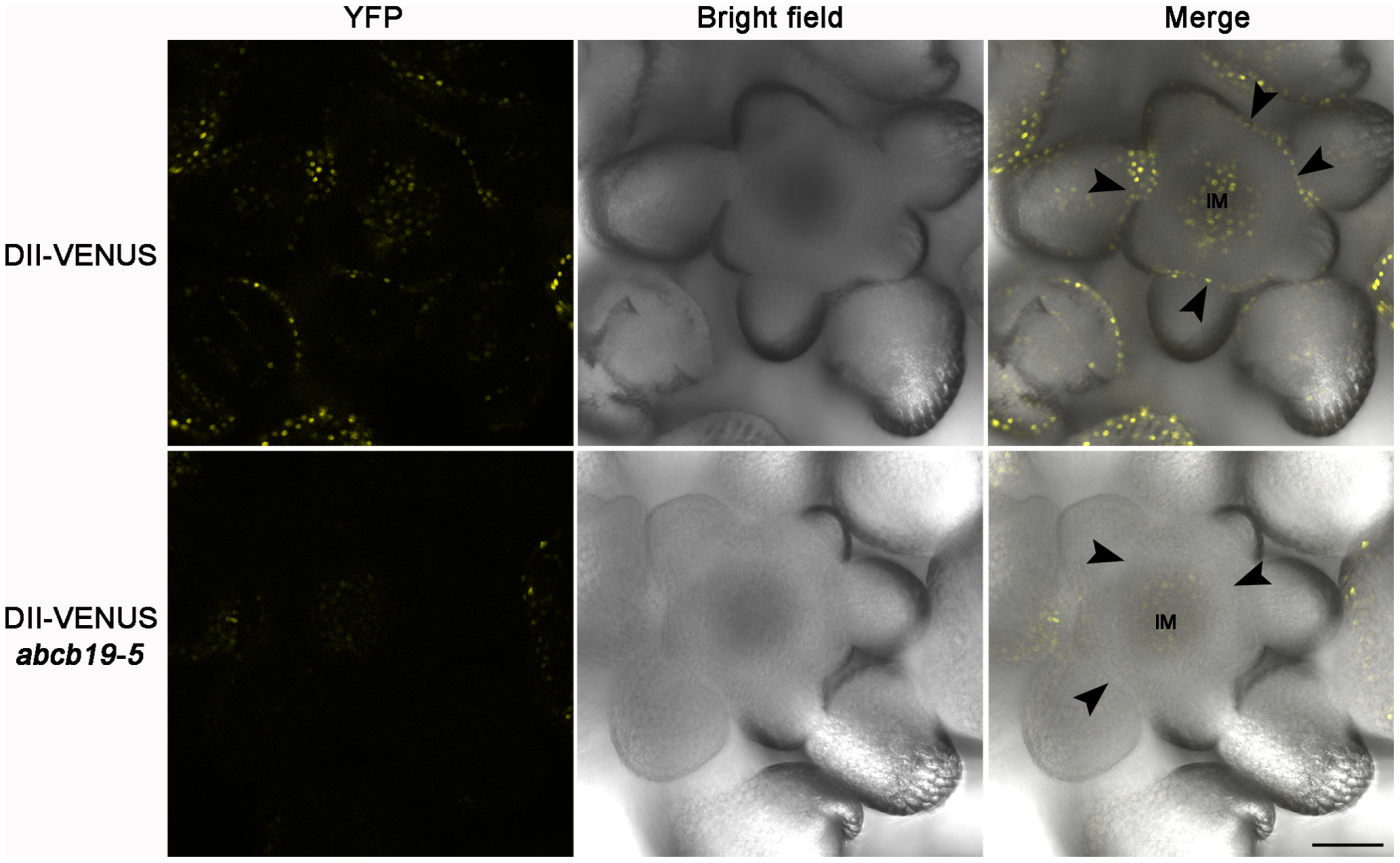
Auxin concentration analysis shown by DII-VENUS in the inflorescence apex. The upper and lower panels are representative of the DII-VENUS fluorescence signal in wild type and *abcb19-5* plants, respectively. Plants were from the F2 population of *abcb19-5*×DII-VENUS. Among the 19 wild type plants, 16 of them had similar (8) or even stronger (8) signal than the upper panel; only 3 plants show a weak signal than that in the upper panel. However, only 5 among 22 *abcb19* plants had similar level of fluorescence to the lower panel; for the other 16 plants, almost no signal was detected in the inflorescence apex; and only one plant show fluorescence signal as strong as that in the upper panel. As a whole, the DII-VENUS signal is obviously reduced in *abcb19*. Arrowheads indicate the organ boundary between inflorescence meristem and floral primordia. IM, inflorescence meristem. Bar  =  50 µm.

Consequently, the DII-VENUS marker was introduced into *abcb19-5*. We found that the overall fluorescence signal was dropped in *abcb19* compared with wild type plants at the inflorescence apex including the inflorescence meristem (IM) and organ boundary region (Figure 3). As a negative indicator of auxin, the reduction of DII-VENUS indicates that the auxin level is increased at the inflorescence apex in *abcb19*, consistent with the abnormal basipetal auxin transport activity in *abcb19*. Thus, by means of DII-VENUS, we show that the endogenous auxin level is increased both in the organ boundary region and in inflorescence meristem (Figure 3).

### *CUC2* and *CUC3* expression is differentially regulated in *abcb19*

Among the genes that function in postembryonic organ boundary separation, *CUC2* and *CUC3* of the NAC family are two well-known, important regulators [8]. And it was indicated that the expression of *CUC2* are inhibited by auxin [18], [19]. To examine the expression of these genes in wild-type and *abcb19* plants, *CUC2::GUS* and *CUC3::GUS* [46] were crossed into *abcb19-5*, respectively.

**Figure 4:**
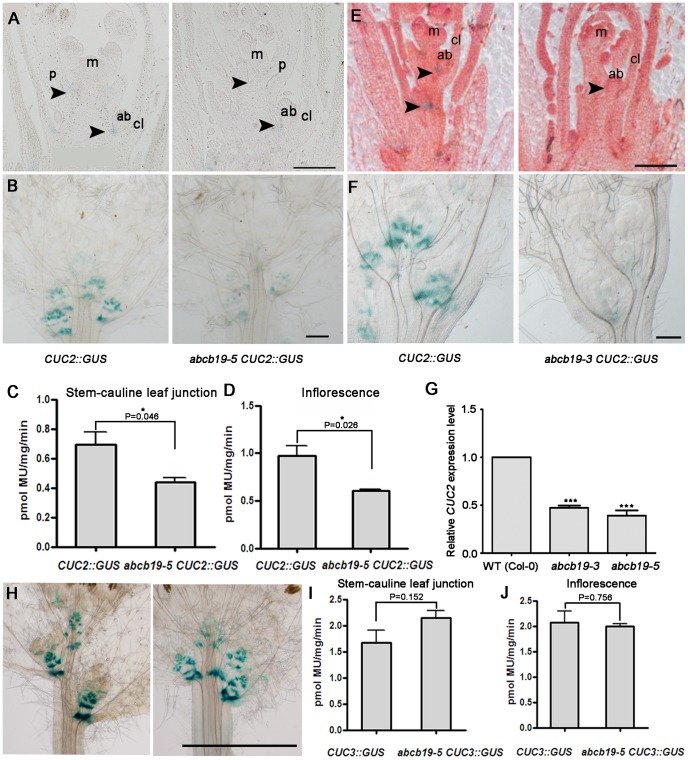
*CUC2/3* expression level in WT and *abcb19-5*. **A:** A longitudinal paraffin section after histological GUS staining of the inflorescence meristem region after the floral transition. Compared with WT, the level of CUC2::GUS activity in *abcb19-5* was obviously reduced. **B:** Histological CUC2::GUS staining of the inflorescence, cauline leaves, and axillary branches done after bolting. The CUC2::GUS level was low in *abcb19-5*. **C** and **D:** β-glucuronidase assay of CUC2::GUS in wild type and *abcb19-5* stem-cauline leaf junctions and inflorescences. For C and D, the values are the mean and standard deviation from three biological replicates (N = 3). The decrease in CUC2::GUS activity in *abcb19-5* was significant (Student's *t*-test, p = 0.046 in C and p = 0.026 in D). * Significantly different, P<0.05. **E** and **F** were similar results as A and B, respectively, except that these are in *abcb19-3*. **G:** Relative expression level of *CUC2* revealed by real-time quantitative-PCR using about 3 mm region including the stem-cauline leaf junction from the secondary branch. *ACTIN2* was used as an endogenous control. Error bars indicate the standard deviation from the three biological replicates. *** Significantly different from the wild type, P<0.001. **H:** Histological CUC3::GUS staining of the inflorescence, cauline leaves, and axillary branches. The CUC3::GUS level in *abcb19-5* was not obviously different from that in WT. **I** and **J:** β-glucuronidase assay of CUC3::GUS in wild-type and *abcb19-5* stem-cauline leaf junctions and inflorescences. For I and J, the values are the mean and standard deviation from three independent biological replicates (N = 3). CUC3::GUS activity in *abcb19-5* was not significantly different from that in WT (Student's *t*-test, p = 0.152 in I and p = 0.756 in J). m, meristem; p, pedicel; ab, axillary bud; cl, cauline leaf. Bar  =  200 µm in **A** and **E**, 1 mm in **B** and **F** and 5 mm in **H**.

The expression patterns of *CUC2::GUS* and *CUC3::GUS* were analyzed at different developmental stages in wild-type and *abcb19* plants. In wild-type, *CUC2::GUS* and *CUC3::GUS* activity was detected at the organ boundary in cotyledons, stem-cauline leaf junctions, and at the boundary of stem-pedicel junctions, consistent with previous *in situ* results [8]. In *abcb19-5*, the *CUC2::GUS* was down-regulated after the plants undergoing the floral transition and after bolting (Figure 4A-B). Furthermore, *CUC2::GUS* activity was reduced by about 37% in both the stem-cauline leaf junctions and inflorescences of *abcb19* plants according to our β-glucuronidase assay results (Figure 4C-D). The CUC2::GUS activity showed similar down-regulation pattern in another allele, *abcb19-3* (Figure 4E-F). However, under the same conditions, the expression of *CUC3* as shown by histological staining and a β-glucuronidase assay was not obviously changed in our experiment (Figure 4H, I, and J). The decrease in the *CUC2* expression in *abcb19* was further confirmed in both *abcb19* alleles by determining the level of *CUC2* mRNA using the real-time quantitative-PCR (q-PCR) (Figure 4G). Thus, mutations in *ABCB19* may specifically affect *CUC2* expression by increased auxin level in the organ boundary region, with no or little effect on *CUC3* expression, during postembryonic growth.

### The genetic relationship between *ABCB19* and *CUC2* or *CUC3* in *Arabidopsis*

*CUC2*, but not *CUC3*, expression was obviously reduced in *abcb19-5*. Given this, we hypothesized that the organ separation defects in *abcb19 cuc3* would be enhanced compared to those in *abcb19*, while the elimination of *cuc2* would not be as efficient as the elimination of *cuc3* in terms of phenotype enhancement.

**Figure 5:**
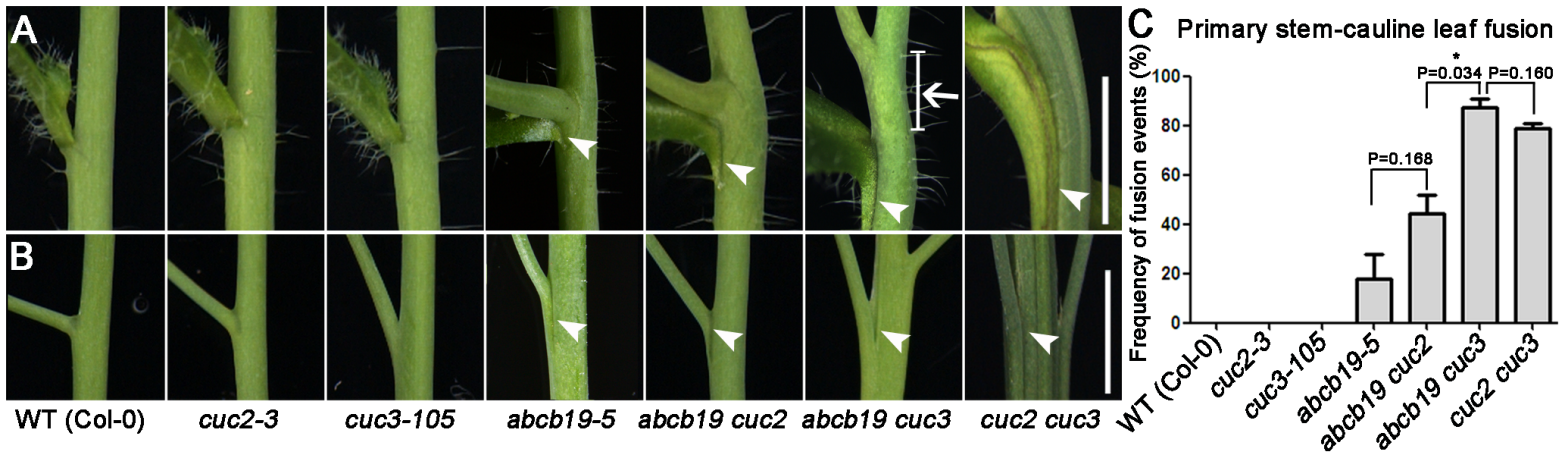
Genetic interaction between *abcb19* and *cuc2*, *cuc3*. **A:** Fusion defects between the primary stem and cauline leaf in *abcb19-5*, *abcb19-5 cuc2*, *abcb19-5 cuc3*, and *cuc2 cuc3*. White arrowheads indicate stem-cauline leaf fusion; white arrow shows the fusion of axillary shoot to the main stem. **B:** Fusion defects between the primary inflorescence stem and pedicel in *abcb19-5*, *abcb19-5 cuc2*, *abcb19-5 cuc3*, and *cuc2 cuc3*. White arrowheads indicate stem-pedicel fusion. **C:** The rate of fusion at primary stem-cauline leaf junctions in different genotypes. At least 30 samples were analyzed for each genotype in every biological replicate. The values represent the mean and standard deviation from two independent biological replicates (N = 2). * Significantly different, P<0.05. Scale bar = 5 mm in **A** and **B**.

To test this hypothesis, we generated *abcb19-5 cuc2-3* and *abcb19 cuc3-105* plants. Consistent with our expectations, *cuc3-105* enhanced the fusion defects seen in *abcb19* dramatically, while *cuc2-3* contributed to the observed defects to a lesser extent (Figure 5A-C). The extent of fusion was greatly enhanced at stem-cauline leaf junctions and inflorescence stem-pedicel junctions in *abcb19-5 cuc3* compared with *abcb19-5* (Figure 5A and B) and fusion of the axillary shoot to the main stem was observed in *abcb19 cuc3*,showing the phenotype equivalence between *abcb19 cuc3* and *cuc2 cuc3* (Figure 5A, shown by an white arrow) [8]; however, the degree of fusion was still less than that seen in *cuc2 cuc3*, due to the residual expression of *CUC2* in *abcb19-5*. *cuc2* enhanced the fusion defects in *abcb19-5* slightly and less effectively than *cuc3* (Figure 5A and B). We next determined the frequency (%) of fusion defects at stem-cauline leaf junctions. In primary stem-cauline leaf junctions, the number of fusion events in *abcb19-5 cuc3-105* was significantly increased compared with *abcb19-5* (Figure 5C); in comparison, the number of fusion events in *abcb19-5 cuc2* was not significantly different from *abcb19-5* (Figure 5C). The rate of fusion in *abcb19 cuc3* was even higher than that in *cuc2 cuc3*, however, the difference was not significant shown by the t-test (Figure 5C).

In general, the lesion of *cuc3* significantly reinforced the fusion defects in *abcb19* in terms of the degree and frequency of fusions, while *cuc2* contributed less. This is largely consistent with the reduced expression of *CUC2* (but not of *CUC3*) in *abcb19* (Figure 4).

### Other organ boundary-specific genes besides *CUC2* may be involved in *ABCB19*-mediated organ separation

As *CUC2* and *CUC3* participate redundantly in postembryonic organ separation, each single mutant shows no obvious fusion defect [8]; thus, only reduction in *CUC2* in *abcb19* does not account for the organ fusion phenotype observed. Since *ABCB19* acts as an auxin transporter, the auxin distribution pattern in *abcb19* is altered obviously (Figure 3). Auxin is such an important regulator of plant development that a number of factors may be changed to varying degrees at the organ boundaries in *abcb19*. Variations in these factors together with the down-regulation of *CUC2* may contribute to the fusion defect observed in *abcb19*.

**Figure 6:**
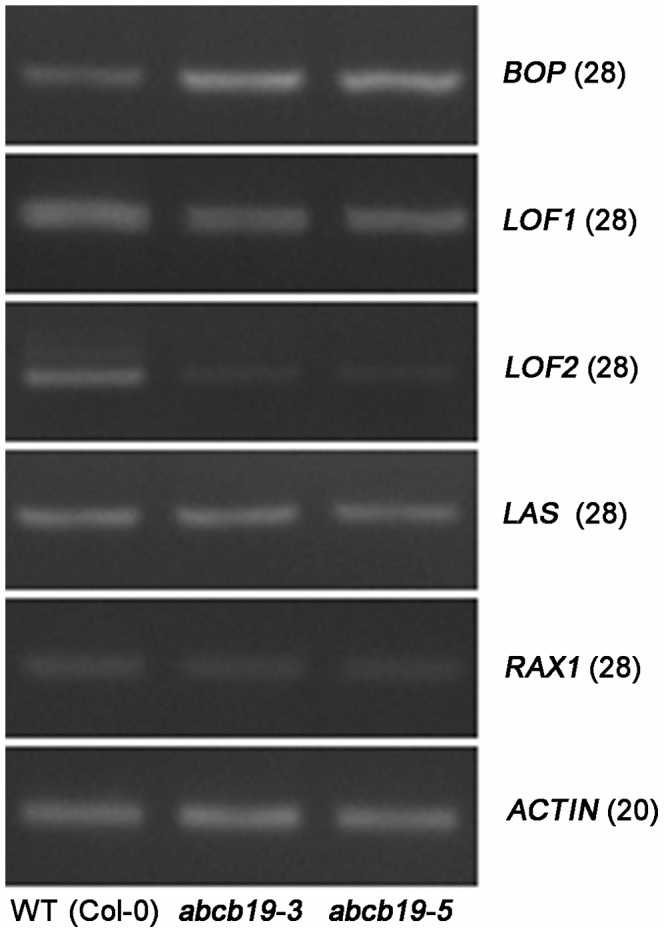
Expression of some organ boundary genes analyzed by semi-quantitative RT-PCR. The numbers labeled on the right are the cycle numbers of the corresponding genes in the RT-PCR. The primer sequences were from the reference [6].

We tested a number of organ boundary-specific factors in *abcb19* by semi-quantitative RT-PCR, and observed that *BOP* was elevated in *abcb19* (the elevated *BOP* expression is similar to the situation in *lof1* [6]); *LOF1* was reduced slightly and *LOF2* was down-regulated obviously; *LAS* and *RAX1* were not distinguishable from the wild type plants (Figure 6). Since it has been shown that the *lof1* knock-out considerably enhances the *cuc2* phenotype [6], the down-regulation of the two *LOFs* in *abcb19* might at least to some extent explain why the *cuc2* phenotype does not match the *abcb19* phenotype.

Therefore, these results demonstrate that *ABCB19*, as an auxin transporter, control a variety of organ boundary genes to guarantee the establishment of the organ boundary.

### *ETT* may function in postembryonic organ separation

**Figure 7:**
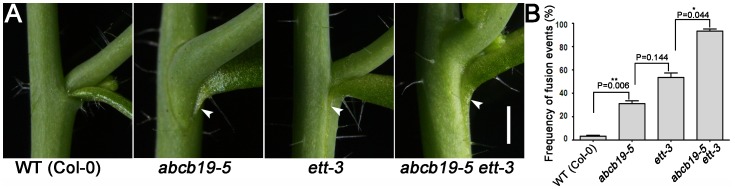
Genetic interaction between *abcb19* and *ett.* **A:** The primary stem-cauline leaf junction fusion seen in *abcb19-5* was enhanced by *ett-3*. White arrowheads indicate stem-cauline leaf fusion. **B:** The rate of fusion in *abcb19* was obviously enhanced by *ett-3*. At least 30 samples were analyzed. The values represent the mean and standard deviation from two independent biological replicates (N = 2). */**Significantly different (p<0.05/p<0.01). Scale bar = 2.5 mm in A.

Auxin functions mainly through *AUXIN RESPONSE FACTORs* (*ARFs*). *ETTIN* (*ETT*)/*ARF3* are reportedly involved in flower development [47], adaxial-abaxial patterning during leaf development [48], and in the vegetative phase change as the target of *trans-acting* (*ta*) *siRNA-ARFs* (*tasiR-ARF*) [49]. We observed that *ett*-*3* showed moderate cauline stem-cauline leaf fusion defects (Figure 7A). When we combined *ett*-*3* with *abcb19-5*, the extent of fusion was dramatically enhanced (Figure 7A). The rate of fusion in *abcb19* was also significantly enhanced by *ett-3* (Figure 7B). This suggests that *ABCB19* participates in a pathway parallel with *ETT* to control postembryonic organ separation.

## Discussion

### *ABCB19* participates in postembryonic organ separation in *Arabidopsis*

ABCB19, as an auxin transporter [24], [29], [31], [32], [39], has been implicated in a multitude of biological processes, including normal growth and development in multiple tissues [24], [39], photomorphogenesis [32], [40], and gravitropic responses [29], [41]. In this study, we generated several lines of evidence showing the novel function of *ABCB19* in postembryonic organ separation based on a mutant identified from our genetic screen. The similar organ separation defects in two alleles of *abcb19* and the appearance of the same defect in F1 plants from a cross between *abcb19-3*/*mdr1-3* and *abcb19-5*, as well as transgenic complementation (Figure 1 and Figure 2), all demonstrate the role of *ABCB19* in organ separation control.

When *ABCB19* is knocked out, the auxin concentration is increased in the boundary region, as is shown by the newly developed DII-VENUS marker (Figure 3). This may result in abnormal cell growth and then the organ fusion defects. We also found that *AUXIN RESPONSE FACTOR-ARF3*/*ETT* is involved in postembryonic organ separation (Figure 7), and that *ABCB19* may participate in a pathway parallel with *ETT* to control postembryonic organ boundary formation.

### *ABCB19* plays a role in organ separation by partially regulating *CUC2* and some other organ boundary genes

Previous studies have indicated that auxin plays a critical role in organ boundary establishment by controlling *CUC* gene expression [18]–[21]. *CUC2* and *CUC3* play redundant roles in postembryonic organ separation [8]. *CUC2* has been frequently reported to be repressed by high auxin concentrations [18], [19]. Notably, we found that the expression of *CUC2* was obviously down-regulated at the postembryonic boundary in *abcb19* compared with wild-type (Figure 4A–G). In contrast, *CUC3* expression was not obviously changed (Figure 4H–J), indicating the differential regulation of these homologs at the transcriptional level by *ABCB19* through the control of auxin distribution. Consistently, it was *cuc3* rather than *cuc2* that enhanced the fusion defects in *abcb19* significantly (Figure 5). Besides *CUC2*, we also found that the some other organ boundary genes, such as *LOF1*, *LOF2*, and *BOP*, were also shown altered expression in *abcb19* (Figure 6). Together, our gene expression and genetic results indicate that *ABCB19* may promote postembryonic organ separation via the regulation of *CUC* and other organ boundary genes, probably through the depletion of auxin at the boundary.

In summary, we demonstrated that the auxin efflux carrier *ABCB19* participates in postembryonic organ boundary specification by partially regulating the NAC family transcription factor *CUC2* and some other organ boundary genes.

## Materials and Methods

### Plant Materials and Growth Conditions

The *Arabidopsis thaliana* plants used in this work were all in the Columbia-0 (Col-0) background. *abcb19-3* (*mdr1-3*) was kindly provided by Dr. Edgar P. Spalding; *abcb19-5*, which carries a T-DNA insertion [50], was cloned by TAIL-PCR. *abcb19-5* was crossed with *CUC::GUSs* to produce *abcb19-5 CUC::GUSs*. In the F2 generation, plants homologous for *abcb19-5* that carried *CUC::GUS* were identified by PCR. In the next generation, thirty seedlings of several different lines were analyzed by GUS staining to identify lines homologous for *CUC::GUS*. *abcb19-5 cuc2-3*, which exhibited an *abcb19*-specific leaf shape and smooth leaf margin (*cuc2-3* phenotype), was first identified by leaf appearance and then by PCR analysis. *abcb19-5 cuc3-105* was characterized by PCR analysis. *abcb19-5 ett-3* was identified by abnormal carpel development (*ett-3*) and the PCR analysis of *abcb19-5*.

Seeds were sterilized in 75% ethanol for 1 min, washed three times with sterile water, kept at 4°C for 2 days to promote germination, and then grown on Murashige and Skoog medium. After 8–10 days of growth chamber (Percival CU36L5) under a cool white fluorescent light (160 µmolm^−2^s^−1^) (16 h of light/8 h of dark, 22°C), the seedlings were transferred to soil and grown in a growth chamber under long-day conditions (16 h of light/8 h of dark) at 22°C and 65% relative humidity.

### Plasmid Construction and Plant Transformation

The full-length CDS of *ABCB19* was amplified from *Arabidopsis* cDNA reverse-transcribed from total seedling RNA using the following primers: *ABCB19*-c-F (5′-CGGGATCCATGTCGGAAACTAACACAACC-3′) and *ABCB19*-c-R (5′-GGGGTACCTCAAATCCTATGTGTTTGAAGC-3′). After sequencing, the *ABCB19* CDS was cleaved with *Bam*HI and *Kpn*I and ligated to the pCAMBIA1300 binary vector under the control of the CaMV *35S* promoter. The construct was then transformed into GV3101 cells and introduced to *abcb19-5* by *Agrobacterium tumefaciens*-mediated floral infiltration as described previously [51].

### RNA Extraction and Real-Time PCR

TotalRNA for was isolated using TRI Reagent Solution (Ambion) according to the manufacturer's handbook. Following digestion with RNase-free DNase (Promega) to eliminate DNA contamination, 3 mg of total RNA were used for reverse transcription (Fermentas). Real-time PCR was carried out using Takara SYBR Premix Ex Taq in a 7500 real-time PCR instrument (Applied Biosystems). Primer information:

ACT2-Q-F, 5′-TCCCTCAGCACATTCCAGCAGAT-3′

ACT2-Q-R, 5′-AACGATTCCTGGACCTGCCTCATC-3′

CUC2- Q-F 5′-GCACCAACACAACCGTCACAG-3′

CUC2- Q-R 5′-GAATGAGTTAACGTCTAAGCCCAAGG-3′

Primers used in the transcript analysis:

P1 5′-GAAGCTGTTGGTTCGGTTTTC-3′

P2 5′-TCAAATCCTATGTGTTTGAAGC-3′

P3 5′-ATGTCGGAAACTAACACAACC-3′

P4 5′-GTAACAGAATCTTTGGGTCTTTC-3′

### GUS Staining

GUS staining and subsequent Paraplast Plus sectioning were performed as described previously [52]. A β-glucuronidase assay was performed according to the protocol of Jefferson [53].

### Confocal Microscopy

Immediately after the plants were bolting, the inflorescences were cut and placed on a slide. Almost all visible buds were cut off and left only the tiny region including the inflorescence meristem. The fluorescent pictures were taken at 40× lens at the excitation of 514 nm on an inverted Zeiss 510 microscope.
